# Brown Adipose Tissue Growth and Development

**DOI:** 10.1155/2013/305763

**Published:** 2013-03-31

**Authors:** Michael E. Symonds

**Affiliations:** Early Life Nutrition Research Unit, Academic Division of Child Health, School of Clinical Sciences, University Hospital, The University of Nottingham, Nottingham NG7 2UH, UK

## Abstract

Brown adipose tissue is uniquely able to rapidly produce large amounts of heat through activation of uncoupling protein (UCP) 1. Maximally stimulated brown fat can produce 300 watts/kg of heat compared to 1 watt/kg in all other tissues. UCP1 is only present in small amounts in the fetus and in precocious mammals, such as sheep and humans; it is rapidly activated around the time of birth following the substantial rise in endocrine stimulatory factors. Brown adipose tissue is then lost and/or replaced with white adipose tissue with age but may still contain small depots of beige adipocytes that have the potential to be reactivated. In humans brown adipose tissue is retained into adulthood, retains the capacity to have a significant role in energy balance, and is currently a primary target organ in obesity prevention strategies. Thermogenesis in brown fat humans is environmentally regulated and can be stimulated by cold exposure and diet, responses that may be further modulated by photoperiod. Increased understanding of the primary factors that regulate both the appearance and the disappearance of UCP1 in early life may therefore enable sustainable strategies in order to prevent excess white adipose tissue deposition through the life cycle.

## 1. Introduction

The study of brown adipose tissue (BAT) biology has always been an exciting and vibrant arena not least because although this tissue is present in comparatively small amounts, it can have a pivotal role in energy balance [1, 2]. BAT is characterised as possessing large amounts of the unique uncoupling protein (UCP) 1 which when activated enables the free-flow of protons across the inner mitochondrial membrane, resulting in the rapid dissipation of chemical energy as heat [1]. Consequently, when maximally activated, BAT can generate up to 300 W/kg of tissue compared with 1 W/kg from most other tissues [3]. This process is regulated primarily by the unmasking of GDP-binding sites located within UCP1 [4, 5] and represents the initial response necessary to ensure rapid heat generation [6]. The primary energy source for this process comes from nonesterified fatty acids that are released from lipid at the same time as UCP1 is activated, usually through activation of the sympathetic nervous system [1]. Despite the control of BAT being well documented from a range of investigations in both small [7] and large mammals [8], it has only been over the past decade following the discovery of the presence of thermogenically active BAT in adult humans that its potential role in a range of homeorhetic processes has been suggested [9]. 

Brown adipose tissue has been the subject of a number of recent reviews which have included a developmental perspective [10] and the potential role it can have on metabolic flux [11] and have largely focused on studies in humans and rodents. The current paper will therefore focus on potential insights that can be gained from also using large animal models of development [12] together with the use of new imaging techniques such as thermal imaging to assess BAT function [13]. Ultimately this may enable a life-course approach to the study of BAT biology in order to provide sustainable interventions aimed at preventing the pronounced loss of BAT with age.

## 2. BAT and Its Role in Obesity

In small animal models defective BAT function is closely associated with increased white fat deposition [16], but in humans although increased body mass index is accompanied with decreased BAT [17, 18], whether this is a cause or consequence remains to be established. The precise contribution of BAT to daily heat production is a contentious issue; it has been calculated that only small amounts of BAT may make a substantial difference to daily energy expenditure with ~60 g of BAT estimated to contribute up to 20% [19] of daily heat production in adult humans. In many genetic studies of obesity, however, the potential role of BAT is largely ignored [20], even in rodent studies that would be expected to impact on BAT function [21].

The developmental regulation of BAT and the extent to which its subsequent loss into adulthood can either be delayed and/or reversed are all factors that could make a significant contribution to overall energy balance [27]. Ideally, these processes need to be considered in view of contemporary lifestyles [28] as a substantial majority of humans now live in an urbanised environment, have a sedentary lifestyle [29], and tend to consume food in a “grazing pattern” through the day rather than have fixed and more modest sized meals 2-3 times a day [28]. All of these factors would be predicted to compromise BAT function and thus contribute to excess white fat deposition although this remains to be fully established. It is also likely that diurnal variations in BAT temperature [30] in addition to more acute changes in response to environmental challenges such as variations in day length [12] will all impact on the ability of BAT to produce heat and therefore energy balance.

## 3. BAT Is Present through the Life Cycle and Has a Different Origin from White Adipose Tissue

The recent rediscovery of BAT in adult humans was the consequence of publications from nuclear medicine describing the symmetrical and differential tissue uptake of ^18^F-fluorodeoxyglucose (FDG) during positron emission tomography (PET) scans undertaken for diagnosis and monitoring of malignant disease. Utilised as an intravenously administered radioactive *g*lucose analogue, FDG is taken up but not metabolized by tissues, and can therefore be used to identify any organ with significant glycolytic activity [31]. As a highly metabolic tissue, BAT exhibits comparable “FDG trapping” accounting for this additional uptake in apparent nontumour sites. Concomitant computed tomography (CT) fusion and guided biopsy of these regions have allowed the localisation of BAT in humans [32, 33]. A number of studies have thus shown that when patients have had some degree of cold exposure prior to undergoing PET-CT, then BAT is readily detectable [15, 34, 35]. Consequently the presence of BAT has now been confirmed in different patient populations from across the world. These include those in Europe [36], North America [37], Australia [38], South Africa [39], Taiwan [40], and China [41] as well as a very small number of healthy volunteers in Europe [15, 34] and Japan [42, 43]. It is now apparent that potentially every human possesses BAT [41], it can be rapidly activated by cold exposure [44], the amount decreases with age [37] and body mass index [18], and it is more likely to be detected in female than male patients [41]. There is, however, considerable variation in potential BAT function which adds to the difficulty in assessing its potential role in overall energy balance regulation [45].

It is not only the distribution of BAT that has been reassessed but also its developmental origin and precursor cell types [46]. Rodent studies have thus established that brown adipocytes are derived from a myogenic lineage, separate entirely from white adipose tissue [47]. Consequently, there are at least three different categories of adipocyte, that is, brown, white, and BRown In whiTE (BRITE), or beige, that may each have separate cell lineages [48]. Furthermore the relative distribution of these individual or mixed cell types varies significantly between each fat depot in the body [49] which may reflect their differential responsiveness to external challenges such as cold exposure [50]. Moreover, at least in mice, genetic variability affects beige, but not BAT, development, suggesting that their regulation is very different during early life [51]. To date, however, the precise role of beige adipocytes in overall energy balance remains to be established as the relative abundance of UCP1 in these cells is substantially lower than “classic” BAT [52], although studies from knockout mice indicate a plethora of regulatory factors [53].

## 4. Maturation at Birth and the Development of BAT

The onset of nonshivering thermogenesis in BAT is a prerequisite for effective adaptation to the cold challenge of the extrauterine environment in a majority of mammals but especially in species that are precocial and do not benefit from huddling with their littermates in order to maintain body temperature [54]. This of course includes humans for whom comparatively large amounts of BAT are present in the newborn, located predominantly around the internal organs, together with interscapular and supraclavicular regions (including discrete depots surrounding the carotid artery and jugular vein) [55]. BAT usually comprises only 2%–4% of birth weight [56, 57] which is not surprising given the high energy costs of fat deposition together with its thermogenic capacity being maximal at birth [58]. Term human infants are also characterised as possessing substantial amounts of subcutaneous white adipose tissue [59] that has an additional insulatory role [60].

There are fundamental differences in the maturation of BAT in the perinatal period between species [61] which reflect both maturity and body composition at birth [62] and can be summarised as follows.

(a) Altricial offspring, such as mice and rats, which are born after a short gestation with an immature hypothalamic-pituitary-adrenal (HPA) axis, maintain their body temperature by the pups huddling together in their nest, rather than by active heatproduction through nonshivering thermogenesis [63]. Consequently their BAT matures postnatally in parallel with maturation of the HPA [64], and maternal-offspring behavioural interactions have a primary role in postnatal temperature control [65].
(b) Precocial offspring such as sheep and humans that are born after a long gestation demonstrate maturation of the HPA prior to birth and are able to rapidly switch on nonshivering thermogenesis following cold exposure to the extra-uterine environment [66]. A failure to switch on BAT, such as following preterm birth, thus impairs heat production and results in hypothermia [67].
(c) One notable exception to the above categories is the pig that lacks BAT [68] as its UCP1 gene is nonfunctional having been disrupted by several mutations [69]. Consequently pigs are entirely dependent on shivering thermogenesis in order to maintain body temperature following cold exposure at birth [14].

## 5. Primary Stages of BAT Development

In fetal sheep, the animal model in which this process has been most intensively studied, adipocytes are clearly visible from mid-gestation when they have a multilocular appearance [70] but do not express UCP1 [71]. They then mature up to term when they are characterised as containing a mixture of unilocular and multilocular cells [72] of which the former are lipid filled, whereas the latter are rich in mitochondria and express UCP1 [70]. The extent to which this is a pure form of BAT as opposed to a mix of beige and white adipocytes remains to be fully clarified although it is now clear that there are at least four distinct stages of adipose tissue development in early life [70]. By mid-gestation when adipose tissue first becomes visible to the naked eye, it has a dense histological cellular structure. Then, close to term, as the depot increases in size, cells with the appearance of both white and brown adipocytes are visible with the latter surrounding the larger, single lipid droplet filled (white) cells [73]. Following birth, a pronounced reduction in the number of white adipocytes occurs coincidently with maximal UCP1 abundance [71]. Finally, a gradual disappearance of brown adipocytes occurs through the postnatal period, culminating in only white adipocytes being discernable by one month of age [70].

The primary phases of fetal/postnatal adipose tissue development, together with the primary regulatory factors, are summarised in Table 1 and include the following. 

### 5.1. A Proliferative Phase in Early-Mid Gestation

This is coincident with the initial appearance of fetal adipose tissue and characterised by rapid cellular multiplication together with maximal expression of KI-67, a marker of cellular proliferation [74]. The perirenal depot thus becomes established from precursor cells, and replication of preadipocytes increases the cell pool and, therefore, depot size. 

 Very few other adipocyte marker genes are highly expressed at this stage [70], with the exceptions being those genes characteristic of cell development such those as the homeobox (HOX) and bone morphogenic protein (BMP) families [75] including HOXA1, HOXC9, and BMP4 and 7 [70] which are indicative of developmental transition. 

### 5.2. Preparative Phase for Thermogenesis Immediately after Birth

The substantial growth of the perirenal-abdominal depot up to term [71] is mediated, in part, by PR domain containing 16 and CCAAT-enhancer-binding protein *β* (C/EBP*β*) which form a transcriptional complex critical for adipogenesis [76]. These genes are probably not involved in promoting the peak in UCP1 expression after birth although this second stage of development is characterised with significant abundance of UCP1 [70]. This process is primarily regulated by the rapid appearance of endocrine stimulatory factors which act to maximise both the amount and thermogenic potential of UCP1 [54, 77]. Notably, gene expression for the long form of the prolactin receptor (PRLR) peaks prior to birth [70], which is in accordance with of its critical role in promoting thermogenesis demonstrated in studies in both small [78] and large [79] mammals. Mice lacking the PRLR have reduced expression of other critical regulators of UCP1 including peroxisome proliferator-activated receptor (PPAR*γ*), peroxisome proliferator-activated receptor c coactivator (PGC1*α*), and the *β*3-adrenergic receptor (*β*3AR) [78]. Concomitant rises in each of these genes prior to birth may all be driven by the PRLR, a role that is confined to birth as the rapid loss of this gene after delivery precedes the decline of UCP1 and continues postnatally [79]. Furthermore, the peak in deiodinase iodothyronine type II (DIO2) [70] would have the potential to provide an endogenous source of triiodothyronine which can activate the thyroid response elements in several genes [80] that are essential for UCP1 function [81]. One such gene, PGC1*α*, has been shown to be the “master regulator” of mitochondrial biogenesis across a range of species [82, 83] and would appear to be a prerequisite for maximising heat production by BAT following cold exposure at birth [84]. At the same time, a peak in gene expression of *β*3AR would facilitate BAT thermogenesis [85] in conjunction with PPAR*α* and PPAR*γ* which can act to reduce adipocyte size and promote mitochondrial biogenesis [86]. The depot size peaks just prior to birth [71], and gene expression of KI-67 declines to basal levels [70] indicating that the replicative period has ceased and cells are committed to terminal differentiation [87]. 

### 5.3. Birth and Nonshivering Thermogenesis

Prior to birth fetal BAT contains a large number of lipid filled cells which become rapidly depleted postnatally, as heat production is maximised [58] and the abundance of the UCP1 peaks [70]. This process is coincident with significant falls in the expression of white adipose tissue marker genes such as adiponectin, leptin, and corepressor receptor-interacting protein 140 (RIP140) [70]. As expected this phase is also characterised by an increase in C/EBP*β* and BMP4 that could be indicative of the start of a change over to a white adipocyte-like phenotype. Notably, there are no further significant changes in the mRNA abundance of any BAT specific genes including cell death-inducing DFFA-like effector A (CIDEA; an established marker of BAT in rodents [88]), together with PRDM16, HOXA1 [70] as well as pyruvate dehydrogenase kinase, isozyme 4 PDK4 [89] that is also highly expressed in other tissues such as skeletal muscle [90].

### 5.4. The Loss of UCP1 and Accumulation of Lipid within the Perirenal-Abdominal Depot

The final phase during early postnatal life coincides with the loss of UCP1 [91] and increasing characteristics of white adipose tissue. Adipocyte size increases and peak expression for a number of genes representative of mature adipocytes occurs, including adiponectin, leptin, and RIP140, together with PPAR*γ*, BMP7, the glucocorticoid receptor (GR2), and HOXC9. Not surprisingly the loss of UCP1 is accompanied by a decline in those genes primarily associated with BAT, that is, PRLR, PGC1*α*, and DIO2. Whilst a linear rise in RIP140 with age is in accord with its repressor action on UCP1 and PGC1*α* activity [92], this process may be facilitated by the concomitant rise in TWIST1 as it is a transcriptional repressor that interacts directly with PGC1*α* to suppress both thermogenic and mitochondrial transcription factors [93]. Gene expression of a number of other genes indicative of a change in adipocyte cell number also peaks at one month of age; however, the primary changes of note are an increase in cell size together with the loss of a BAT phenotype [70]. Continuing differentiation of white adipocytes is possible as indicated by the rise in gene expression of BMP4 [70], although BMP7, a BAT differentiation marker, is also evident [94]. Other genes which may regulate postnatal adipose tissue growth include the GR2, which promotes UCP1 action in the fetal sheep [95] and gene expression rises in parallel with increased white fat mass through postnatal and juvenile life [96]. It is possible that this depot is comprised of beige adipocytes that have the capacity to respond to an appropriate challenge [70] although this remains to be established.

## 6. Rapid Appearance of Endocrine Stimulatory Factors and the Onset of Nonshivering Thermogenesis in BAT

**Figure 1:**
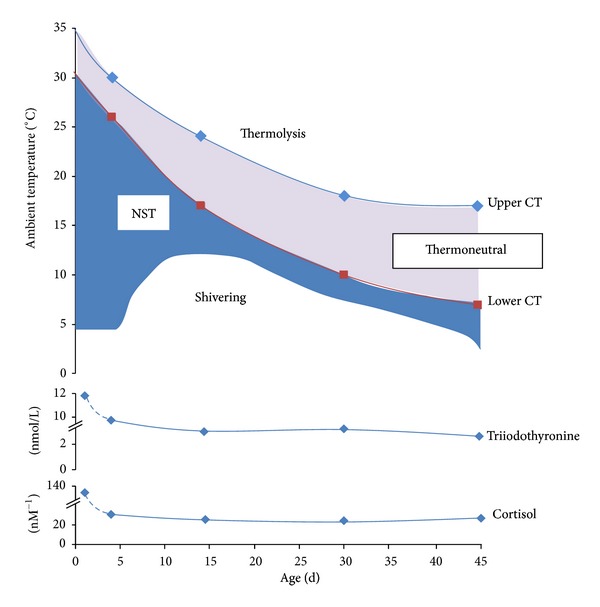
Summary of the metabolic and endocrine changes that occur from birth to 45 days of postnatal life in the sheep as brown adipose tissue is lost and shivering replaces nonshivering thermogenesis (NST) as the primary response to cool exposure. Adapted from Symonds et al. [104]. CT: critical temperature.

The plethora of endocrine changes which occur at birth following intense stimulation of the hypothalamic-pituitary-thyroid and adrenal axes is essential for the rapid activation of nonshivering thermogenesis [97]. This process has been extensively studied in the newborn sheep for which impaired BAT thermogenesis not only compromises the onset of breathing but also results in hypothermia that is ultimately life-threatening [98]. A large number of endocrine factors have the potential to activate BAT around the time of birth and include catecholamines [99], thyroid hormones [100], cortisol [101], leptin [102], and prolactin [79]. The secretion and plasma concentrations of a majority of these hormones then decline over the first few days and weeks of life [54, 103] as shivering replaces nonshivering thermogenesis as the dominant response to cold exposure [104] (Figure 1). It thus appears that it is only possible to very transiently promote the reappearance of BAT during the postnatal period at least in a precocial species [83].

Given the multiple endocrine factors described above which can act either alone or in combination, to promote both the rapid appearance of UCP1 loss of lipid [105], and there is some duplication of roles within this overall process. For example, manipulation of cortisol status in the late gestation ovine fetus has reciprocal effects on thyroid hormone concentrations that may be as important as changes in plasma cortisol on the downstream effects on UCP1 [95]. These findings emphasise the multiple endocrine regulation of BAT in the newborn and are thus in accord with the plethora of knock-out studies in mice which similarly demonstrate a large number of factors able to regulate UCP1 abundance and function [7, 106]. One challenge is to now identify those which can be chronically stimulated in order to maintain and/or reactivate physiologically significant amounts of BAT in later life. 

## 7. Other Organs Potentially Contributing to Nonshivering Thermogenesis at Birth

Birth represents a period of rapid muscular activity and is associated with a dramatic rise in muscle oxygenation [107] and cardiac function [108]. In precocial mammals such as sheep, it is therefore accompanied with an increase in voluntary muscular activity, and with the onset of shivering thermogenesis [109] which is, in turn, dependent on both the BAT function [110] and the magnitude of thermal challenge [104]. The process of adaptation at birth may, therefore, provide further currently unexplored insights into the crosstalk between different muscle and fat depots, together with the ability of other organs to promote BAT function in early life. Currently this has been difficult to establish in small animal models because of the dominant role of UCP1; however when it is ablated, other factors are beginning to be identified in skeletal muscle which include sarcolipin, a recently described regulator of the sarco/endoplasmic reticulum Ca^2+^−ATPase [111].

There is also increasing evidence that a number of other tissues and endocrine factors can promote BAT function although their importance at birth remains unknown. For example, in rats, the postnatal maturation of BAT has been shown to relate to the onset of feeding and initiation of hepatic function, mediated by the release of fibroblast growth factor (FGF)21 [112]. This factor has also been shown to have a physiological role in adipose tissue of adult mice in which gene expression is increased by chronic cold exposure, in contrast to its suppression in the liver [113]. The potential link between developmental changes in hepatic FGF21 production, feeding, and UCP1 has not yet been examined in large mammals. It is also unlikely that under normal physiological conditions the other major UCPs, UCP2 and UCP3, have a major role in heat generation that is comparable to UCP1 [114].

## 8. Postnatal Adaptation to the Extrauterine Environment and the Loss of BAT

The direct contribution that BAT makes to overall energy balance is clearly indicated by the very high rates of oxygen consumption that are seen in the newborn and are seldom matched in later life [104]. This high rate of heat production occurs in the absence of any visible signs of shivering [104] and is dependent on the magnitude of thermal challenge and nutritional status [115]. It is also closely linked to functional measurements of BAT such as its thermogenic index [116]. In those species such as sheep in which BAT is lost and/or replaced by white [91] or beige adipose tissue [70] over the first few weeks of life, there is a parallel decline in oxygen consumption and increased dependency on shivering thermogenesis to respond to acute cold challenges [104]. As summarised in Figure 1, these changes are paralleled by a decline in plasma cortisol and thyroid hormones, thereby confirming that it is not only the recruitment of BAT [117] at birth but its subsequent maintenance which is thyroid dependent [54]. Moreover, accelerating the rate of decline in plasma thyroid hormones over the postnatal period by either blocking their rate of secretion [118] and/or warm acclimation promotes the loss of UCP1 and enhances white adipose tissue deposition [118, 119]. At the same time these animals are more dependent on shivering thermogenesis which is ultimately an inefficient process due to disruption of the boundary layer of air insulating the animal [120]. Ultimately hypothyroidism during postnatal life results in a failure to thrive impaired thermoregulation and leads to unexpected death [104, 121].

The extent to which there are specific beige depots or skeletal muscle that possesses UCP1 in the newborn remains to be fully established. Given that BAT is so widely distributed at this stage of life [55, 122], it is unlikely that such discrete depots have a significant impact on heat production especially as the relative abundance of UCP1 is so much lower than classical BAT [49]. Interestingly, however, in view of the common origin of brown adipocytes and skeletal muscle [47], a close link between functional BAT and muscle volume has recently been suggested in children and adolescents [123]. To date, this relationship has not been studied in very early life, although it has been shown that deletion of myostatin, a negative regulator of skeletal muscle mass, in mice prevents diet induced obesity and not only promotes skeletal muscle growth but also promotes the appearance of beige cells [124]. These findings may be relevant given the recent suggestion that, in adults, muscular activity can directly impact on adipose tissue function [125]. This effect appears to be mediated by an increased rate of production of a newly discovered hormone, irisin, which is a membrane protein cleaved from FNDC5 [125], although this role has been questioned [126]. Surprisingly, irisin promotes the expression of UCP1 in white but not BAT at least in adults [125]. The extent to which it could have a thermogenic influence on muscle, as well as BAT, has not been examined. Skeletal muscle does possess the UCP homolog UCP3 [127] that is highly abundant in the muscle and adipose tissue of neonatal piglets [128], and in mice overexpression of this protein protects against obesity [129]. At the same time MED13, a subunit of the Mediator complex, which controls the transcription of nuclear hormone receptors can regulate energy metabolism in cardiac muscle and impact on the appearance of BAT [130].

## 9. The Heart, BAT, and Early Life Nutritional Programming

In sheep, for which pericardial adipose tissue is the second most abundant BAT depot in the newborn [72], this appears to show similar developmental changes to the perirenal depot [131]. It is well established that the maternal dietary environment has pronounced effects on growth, development, and endocrine sensitivity of perirenal adipose tissue during early life [132–134] and now it appears that a similar situation pertains to the pericardial depot [131]. In this regard, suboptimal maternal nutrition over the final month of gestation coincident with the greatest increase, in absolute terms, of fetal total and organ weights, substantially reduces UCP1 abundance in BAT surrounding the heart [131]. This was accompanied with reduced expression of *β*3ADR at birth and one month of age, whilst gene expression of DIO2 was only reduced in the newborn. The *β*3ADR receptor promotes proliferation and differentiation of brown adipocytes, inhibits apoptosis and controls the process of nonshivering thermogenesis, by upregulating fatty acid oxidation and activating DIO2 [1]. The effect of a suboptimal diet on fetal adipose tissue development is however confined to these two genes, for example, the expression of transglutamiase 2 (TGM2) and potassium channel, subfamily member 3 (KCNK3), two genes known to have 4-fold higher expression in BAT than in white adipose tissue in humans [135], was unaffected [131]. These genes were, however, highly expressed in perirenal adipose tissue of young sheep and although expression declined by 30 days of age, it was parallel with changes in both UCP1 and PGC1*α* [131]. Gene expression of the transcription factor, BMP7, which regulates the differentiation of preadipocytes into mature BAT cells [75], remained unchanged [131], suggesting that, as with the perirenal depot, although brown adipocytes disappear [70], preadipocytes may be retained with the potential to develop into brown adipocytes.

UCP1 has also been shown to be present in epicardial adipose tissue of human adults [136] where it may have a range of functions. These include protection of the myocardium from severe hypothermia which can precipitate potentially fatal arrhythmias [136] and paracrine effects on cardiovascular function [137] or act as a plasma lipid-clearing organ protecting the heart from hypertriglyceridemia [138]. Although BAT development in pericardial adipose tissue of newborns was effected by maternal nutrition in late gestation, these did not persist with age and were not accompanied by alteration of genes predominantly expressed in white adipose tissue, indicating that the response is restricted to the newborn period. Adaptations within pericardial adipose tissue may persist into adulthood and thus have long-term consequences [139]. 

A potential link between muscle and BAT development has been further highlighted from studies focused on the heart [140]. The heart, acting through cardiac natriuretic peptides, can regulate BAT thermogenesis in adult mice [141]. This response appears to be mediated by ventricular or brain natriuretic peptide (BNP) which promotes UCP1 expression, although this effect is potentially greater in inguinal white adipose tissue, compared to interscapular BAT. Given the relatively high expression of UCP1 in human [136], ovine [142], and mouse [49] epicardial fat, this could be an important therapeutic target. 

## 10. Do Beige Adipocytes or Other Organs/Tissues Possessing UCP1 Contribute Significantly to Whole Body Energy Balance?

The theoretical contribution of BAT to daily energy expenditure ranges from a maximum estimate of 20% [143] to a basal figure of 5% [15] based on an estimated total BAT mass of up to ~60 g in an adult [143]. It is clear that it is not only the quantity of BAT that is important but also the absolute amount of UCP1 present together with its ability to be activated [144]. The major depot of BAT in humans throughout the life cycle is clearly the supraclavicular depot [144] as emphasised by the rapidity with which it can be activated [44] and its colocation with hibernomas that are defined as being primarily BAT [145]. Interestingly under such conditions of excessive BAT growth, no effects appear to be seen on temperature regulation either before or after surgical removal [145, 146], suggesting that UCP1 abundance is low and/or it is inactive in this abnormal tissue.

**Figure 2:**
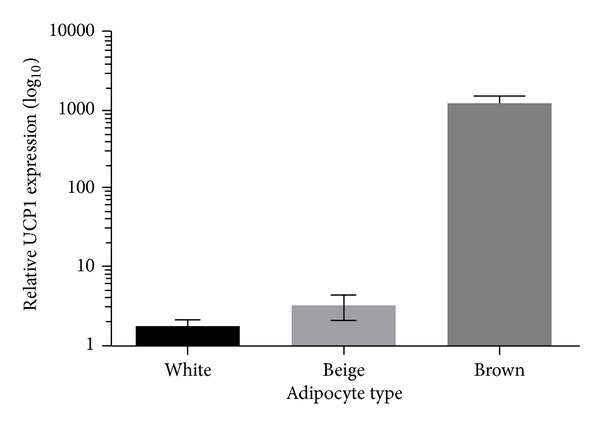
Summary of the differences in relative gene expression for uncoupling protein (UCP) 1 between white subcutaneous adipose tissue and either beige or brown depot of supraclavicular adipose tissue, classified according to low (i.e., <10) or high (i.e., >100) relative UCP1 gene expression. Adapted from Virtanen et al. [15].

The capacity for heat production within the supraclavicular region is high in children and then declines into adulthood [44]. This process, or adaptation, is in accord with the suggestion that ultimately BAT within the supraclavicular region becomes beige [48]. As illustrated in Figure 2, the process by which BAT becomes beige can vary considerably between individuals. It may be that only when UCP1 abundance declines below a critical set-point and is accompanied by significant lipid and thus white adipocyte infiltration that it then resembles a beige depot. The study by Wu et al. [48] therefore suggests that adipocytes isolated from the main functional depot of “brown” adipose tissue in adult humans, that is, supraclavicular, eventually become beige rather than remaining “classical” BAT. This conclusion is based in part on the differences and/or similarities in the relative expression of beige or brown-selective genes between white and “brown” depots. However, it is apparent from Figure 2 that there are two different populations of subjects included, one in which UCP1 was very high (i.e., characteristic of a “brown” depot) and the other in which it was low (i.e., characteristic of a “beige” depot). It would thus be fascinating to know whether the relative abundance of UCP1 gene in these subjects was related to their metabolic response as measured to cold exposure [34]. The actual capacity for either beige adipocytes or skeletal muscle cells possessing UCP1 to generate the same amount of heat as classical BAT in which UCP1 is several fold higher [27] may thus be minimal even when maximally stimulated.

## 11. Dietary Induced Thermogenesis (DIT) and BAT Function

Once the newborn has established continuous breathing and activated nonshivering thermogenesis, it is essential that feeding is commenced in order to prevent excessive depletion of its endogenous energy supplies in the form of both lipid [147] and glycogen [14, 54]. Furthermore, in the postnatal sheep the gradual loss of BAT is accompanied with a reduction in DIT [115]. However, the extent to which BAT contributes to DIT in adults remains controversial [41, 148], although FDG uptake in BAT of mice is clearly enhanced with feeding [149]. Using thermal imaging, the potential thermogenic effects of individual food ingredients are beginning to be investigated. This has demonstrated a significant thermogenic effect of drinking milk in young children which results in up to 0.7°C rise in BAT temperature [150], thus indicating a role in DIT. 

**Figure 3:**
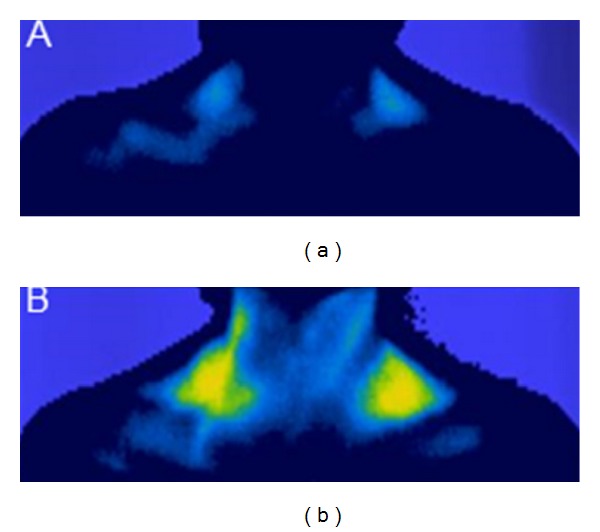
Representative example of thermal images [31] showing the change in temperature of BAT located within the supraclavicular region of a prepubertal 13-year-old child having eaten 25 g of organic porridge oats mixed with 70 mL of full fat milk and 8 g of brown sugar. (a) Preprandial control and (b) 5 minutes after-prandial, accompanied with a mean increase in temperature of the supraclavicular region of 0.9°C.

Confirmation of a potential role of dietary intake is further illustrated in Figure 3 in which an example of thermal images showing the temperature of BAT located within the supraclavicular region of a pre-pubertal 13-year-old child before and after consumption of breakfast demonstrates a mean increase in temperature of the supraclavicular region of 0.9°C. It is likely that the macronutrient composition determines the magnitude of this response [151] that has been shown in adults to be promoted by protein and reduced by fat [152]. The only other study showing a dietary effect on BAT comes from Japan which has suggested that capsinoids (nonpungent capsaicin analogs) have a modest thermogenic effect [153]. This response, however, appeared to be confined to those individuals that were defined as being BAT positive following the assessment of FDG uptake within BAT following cold exposure. Whether or not this directly relates to nonfunctional BAT or is simply a limitation of the FDG technique [144] remains to be established.

The potentially divergent effects of specific endocrine challenges on BAT compared with cold exposure have been highlighted by the failure to detect any effect of administration of sympathomimetics on BAT, as assessed using FDG in adult humans which were all shown to be responsive to cold exposure [154, 155]. This is despite a comparable increase in total heat production that was accompanied by raised fat oxidation that is the main metabolic substrate for nonshivering thermogenesis in BAT. These compounds do, however, have very different metabolic effects to cold exposure which include the onset of metabolic acidosis [154] which could compromise BAT function [156]. Alternatively, the dose of drug administered could be important, together with body weight, as lean males have recently been shown to respond to a higher dose of ephedrine [157]. Furthermore, insulin also acts differently to cold exposure on human BAT, and although it has no thermogenic effect on BAT it does promote glucose uptake [35]. It is therefore likely that multiple endocrine responses following cold exposure are responsible for stimulating BAT thermogenesis, as seen at birth when UCP1 is maximally activated [58].

## 12. Can BAT Be (Re)activated in Adulthood?

To date, all studies investigating the reactivation of BAT have been conducted on rodents in which it is becoming increasingly apparent that different control mechanisms and sensitivities exist between brown and white fat depots as summarised in Table 2. These experiments have adopted a common approach of either global or organ specific gene manipulation which invariably has a substantial impact on energy balance in rodents. In this context knockout of BMP8B, a protein that is highly expressed in the testis as well as BAT results in reduced UCP1 function, due to compromised activity of the sympathetic nervous system [22]. This effect appeared to be mediated centrally in conjunction with changes in AMP-activated protein kinase, although potential effects on white or beige adipose depots were not examined. Another protein recently suggested to represent a therapeutic target to promote BAT function was the mediator of cell signal transduction, scaffold protein p62 [23]. When this was specifically knocked out in adipocytes a global reduction in UCP1 was observed, that is, in brown, white, and beige depots. Conversely conditional knockdown of the phosphatase and tensin homolog (PTEN) in the Myf+ lineage, which negatively regulates phosphatidylinositol 3-kinase activity, surprisingly results in larger brown and white adipocytes [24]. This suggests that both types of cells have an Myf+ origin but under normal conditions the lipogenic aspect of the pathway could be limited. 

SERTA domain containing 2 (TRIP-Br2) is a further novel factor that has been shown to be positively linked with white mass, although this relationship is much stronger in men than women [25]. Knockout studies in rodents indicate a stimulatory response in BAT, thus suggesting that it normally, promotes fat deposition by inhibiting lipolysis, thermogenesis, and oxidative metabolism. Similarly, gene deletion of retinaldehyde dehydrogenase 1a which normally inhibits the browning of white adipose tissue has beneficial metabolic effects [26]. Despite these elegant studies demonstrating even more potential therapeutic targets to promote UCP1 abundance, it should be noted that they are all conducted in mice maintained within a comparatively cool environmental temperature of 21–23°C and kept under a fixed 12 h light and 12 h day photoperiod. These experimental constraints may ultimately limit the translational relevance of these important findings which in humans the pronounced effects of age, lifestyle, and environment on energy balance are substantial [28].

In addition to the thermal, nutritional, and related environmental stimuli, BAT is influenced by a range of other factors including genotype for which *β*3-adrenergic receptor polymorphisms have been identified in both humans [158] and sheep [159]. These can have a profound effect on postnatal survival in sheep [159] but its influence in humans is less obvious. In a small Japanese cohort in which three different polymorphisms were identified, the distribution of BAT positive subjects was only associated with individual genotypes when groups were subdivided with age [158]. Genotype can influence BAT function [144] and the use of thermal imaging offers the potential to assess this relationship in large populations of known genetic constitution.

Consequently, as comparable noninvasive and safe methods for detecting BAT on a population-wide basis are established, significant progress on the interaction between genotype, age, diet, and environment can be made [13]. These types of study are a real possibility and predicted to open up a range of new horizons in adipose tissue biology over the next decade [17]. This could mean that a more direct relationship between body weight regulation and BAT function is finally established throughout the life cycle in humans.

## Figures and Tables

**Table 1:** Summary of the main developmental changes in adipose tissue during early life.

Stage of development	Proliferative phase	Preparatory phase	Thermogenic phase	Lipogenic phase
Primary adipose tissue characteristics	Preadipocyte	Brown adipose tissue	Brown adipose tissue	White adipose tissue
Function	Cellular multiplication necessary to form adipose tissue depot	Acquisition of large amounts of uncoupling protein 1	Rapid activation of uncoupling protein 1 in order to prevent hypothermia	Lipid deposition and storage
Most abundant gene	Antigen identified by monoclonal antibody ki-67	Long form of prolactin receptor	Uncoupling protein 1	Leptin

**Table 2:** Summary of recent targets for gene manipulation studies designed to impact on brown fat function in adult rodents maintained in a fixed thermal and photoperiodic environment.

Target function, based on brown fat function in the knock out	Effect on brown adipose tissue	Effect of white adipose tissue	Phenotype	Primary mechanism	Reference
Inhibitory					

Bone morphogenetic protein (BMP8B) knockout	Normal but reduced thermogenic activity, most apparent during cold exposure	Not examined	Lower body temperature, increased body mass, and an adaptation amplified with consumption of an HFD	Modulates SNS activity within BAT	[22]
Scaffold protein p62, adipocyte specific knockout	Reduced activity and responsiveness to norepinephrine	Reduced UCP1 within inguinal	Increased body weight and fat mass and an adaptation reduced when fed an HFD	Acts specifically on mitochondrial function in brown adipocytes and thus thermogenesis	[23]

Stimulatory					

Phosphatase and tensin homolog, conditional knockdown	Increased adipocyte cell size	Increased adipocyte cell size	Despite similar body mass, WAT distribution disorder is apparent	Both brown and white cells may have Myf5+ origins	[24]
SERTA domain containing 2 (TRIP-Br2) knock out	Increased thermogenic activity and cold responsiveness	Decreased adipocyte cell size	Improved glucose homeostasis and ability to maintain body temperature during cold exposure	Modulates fat storage through inhibition of lipolysis, thermogenesis, and oxidative metabolism	[25]
Retinaldehyde dehydrogenase 1a, knockout	None	Increased UCP1 with a greater response in perigonadal compared with inguinal	Improved glucose homeostasis and ability to maintain body temperature during cold exposure	Inhibits the browning of WAT	[26]

BAT: brown adipose tissue; HFD: high fat diet; SNS: sympathetic nervous activity; WAT: white adipose tissue.

## References

[1] Cannon B, Nedergaard J (2004). Brown adipose tissue: function and physiological significance. *Physiological Reviews*.

[2] Smith RE, Horwitz BA (1969). Brown fat and thermogenesis. *Physiological Reviews*.

[3] Power GG (1989). Biology of temperature: the mammalian fetus. *Journal of Developmental Physiology*.

[4] Heaton GM, Nicholls DG (1977). The structural specificity of the nucleotide-binding site and the reversible nature of the inhibition of proton conductance induced by bound nucleotides in brown-adipose-tissue mitochondria. *Biochemical Society Transactions*.

[5] Nicholls DG, Locke RM (1984). Thermogenic mechanisms in brown fat. *Physiological Reviews*.

[6] Trayhurn P, Ashwell M, Jennings G, Richard D, Stirling DM (1987). Effect of warm or cold exposure on GDP binding and uncoupling protein in rat brown fat. *American Journal of Physiology*.

[7] Kozak LP, Koza RA (2010). The genetics of brown adipose tissue. *Progress in Molecular Biology and Translational Science*.

[8] Symonds ME, Pope M, Sharkey D, Budge H (2012). Adipose tissue and fetal programming. *Diabetologia*.

[9] Nedergaard J, Bengtsson T, Cannon B (2007). Unexpected evidence for active brown adipose tissue in adult humans. *American Journal of Physiology*.

[10] Ravussin E, Galgani JE (2011). The implication of brown adipose tissue for humans. *Annual Review of Nutrition*.

[11] Bartelt A, Heeren J (2012). The holy grail of metabolic disease: brown adipose tissue. *Current Opinion in Lipidology*.

[12] Symonds ME, Sebert SP, Budge H (2010). Nutritional regulation of fetal growth and implications for productive life in ruminants. *Animal*.

[13] Symonds ME, Budge H (2012). How promising is thermal imaging in the quest to combat obesity?. *Imaging in Medicine*.

[14] Mellor DJ, Cockburn F (1986). A comparison of energy metabolism in the new-born infant, piglet and lamb. *Quarterly Journal of Experimental Physiology*.

[15] Virtanen KA, Lidell ME, Orava J (2009). Functional brown adipose tissue in healthy adults. *The New England Journal of Medicine*.

[16] Hamann A, Flier JS, Lowell BB (1996). Decreased brown fat markedly enhances susceptibility to diet-induced obesity, diabetes, and hyperlipidemia. *Endocrinology*.

[17] Vijgen GH, Bouvy ND, Teule GJ (2012). Increase in brown adipose tissue activity after weight loss in morbidly obese subjects. *The Journal of Clinical Endocrinology & Metabolism*.

[18] Vijgen GHEJ, Bouvy ND, Teule GJJ, Brans B, Schrauwen P, van Marken Lichtenbelt WD (2011). Brown adipose tissue in morbidly obese subjects. *PLoS ONE*.

[19] Ouellet V, Labbe SM, Blondin DP (2012). Brown adipose tissue oxidative metabolism contributes to energy expenditure during acute cold exposure in humans. *The Journal of Clinical Investigation*.

[20] Parks Brian W, Nam E, Org E (2013). Genetic control of obesity and gut microbiota composition in response to high-fat, high-sucrose diet in mice. *Cell Metabolism*.

[21] Fromme T, Klingenspor M (2011). Uncoupling protein 1 expression and high-fat diets. *American Journal of Physiology*.

[22] Whittle AJ, Carobbio S, Martins L (2012). BMP8B increases brown adipose tissue thermogenesis through both central and peripheral actions. *Cell*.

[23] Muller TD, Lee SJ, Jastroch M (2013). P62 Links beta-adrenergic input to mitochondrial function and thermogenesis. *The Journal of Clinical Investigation*.

[24] Sanchez-Gurmaches J, Hung CM, Sparks CA, Tang Y, Li H, Guertin DA (2012). PTEN loss in the Myf5 lineage redistributes body fat and reveals subsets of white adipocytes that arise from Myf5 precursors. *Cell Metabolism*.

[25] Liew CW, Boucher J, Cheong JK (2013). Ablation of TRIP-Br2, a regulator of fat lipolysis, thermogenesis and oxidative metabolism, prevents diet-induced obesity and insulin resistance. *Nature Medicine*.

[26] Kiefer FW, Vernochet C, O'Brien P (2012). Retinaldehyde dehydrogenase 1 regulates a thermogenic program in white adipose tissue. *Nature Medicine*.

[27] Symonds ME, Budge H, Perkins AC, Lomax MA (2011). Adipose tissue development—impact of the early life environment. *Progress in Biophysics and Molecular Biology*.

[28] Symonds ME, Sebert S, Budge H (2011). The obesity epidemic: from the environment to epigenetics—not simply a response to dietary manipulation in a thermoneutral environment. *Frontiers in Epigenomics*.

[29] Jacobson SH, King DM, Yuan R (2011). A note on the relationship between obesity and driving. *Transport Policy*.

[30] Blessing W, Mohammed M, Ootsuka Y (2012). Heating and eating: brown adipose tissue thermogenesis precedes food ingestion as part of the ultradian basic rest-activity cycle in rats. *Physiology & Behavior*.

[31] Hany TF, Gharehpapagh E, Kamel EM, Buck A, Himms-Hagen J, von Schulthess GK (2002). Brown adipose tissue: a factor to consider in symmetrical tracer uptake in the neck and upper chest region. *European Journal of Nuclear Medicine*.

[32] Cohade C, Osman M, Pannu HK, Wahl RL (2003). Uptake in supraclavicular area fat (“USA-Fat”): description on 18F-FDG PET/CT. *Journal of Nuclear Medicine*.

[33] Yeung HWD, Grewal RK, Gonen M, Schöder H, Larson SM (2003). Patterns of 18F-FDG uptake in adipose tissue and muscle: a potential source of false-positives for PET. *Journal of Nuclear Medicine*.

[34] van Marken Lichtenbelt WD, Vanhommerig JW, Smulders NM (2009). Cold-activated brown adipose tissue in healthy men. *The New England Journal of Medicine*.

[35] Orava J, Nuutila P, Lidell ME (2011). Different metabolic responses of human brown adipose tissue to activation by cold and insulin. *Cell Metabolism*.

[36] Au-Yong ITH, Thorn N, Ganatra R, Perkins AC, Symonds ME (2009). Brown adipose tissue and seasonal variation in humans. *Diabetes*.

[37] Cypess AM, Lehman S, Williams G (2009). Identification and importance of brown adipose tissue in adult humans. *The New England Journal of Medicine*.

[38] Lee P, Greenfield JR, Ho KKY, Fulham MJ (2010). A critical appraisal of the prevalence and metabolic significance of brown adipose tissue in adult humans. *American Journal of Physiology*.

[39] Perkins AC, Mshelia DS, Symonds ME, Sathekge M (2013). Prevalence and pattern of brown adipose tissue distribution of 18F-FDG in patients undergoing PET-CT in a sub-tropical climatic zone. *Nuclear Medicine Communications*.

[40] Huang YC, Hsu CC, Huang P (2011). The changes in brain metabolism in people with activated brown adipose tissue: a PET study. *NeuroImage*.

[41] Nedergaard J, Bengtsson T, Cannon B (2010). Three years with adult human brown adipose tissue. *Annals of the New York Academy of Sciences*.

[42] Saito M, Okamatsu-Ogura Y, Matsushita M (2009). High incidence of metabolically active brown adipose tissue in healthy adult humans: effects of cold exposure and adiposity. *Diabetes*.

[43] Yoneshiro T, Aita S, Matsushita M (2011). Age-related decrease in cold-activated brown adipose tissue and accumulation of body fat in healthy humans. *Obesity*.

[44] Symonds ME, Henderson K, Elvidge L (2012). Thermal imaging to assess age-related changes of skin temperature within the supraclavicular region co-locating with brown adipose tissue in healthy children. *Journal of Pediatrics*.

[45] van Marken Lichtenbelt WD, Schrauwen P (2011). Implications of nonshivering thermogenesis for energy balance regulation in humans. *American Journal of Physiology*.

[46] Cannon B, Nedergaard J (2012). Cell biology: neither brown nor white. *Nature*.

[47] Seale P, Bjork B, Yang W (2008). PRDM16 controls a brown fat/skeletal muscle switch. *Nature*.

[48] Wu J, Bostrom P, Sparks LM (2012). Beige adipocytes are a distinct type of thermogenic fat cell in mouse and human. *Cell*.

[49] Walden TB, Hansen IR, Timmons JA, Cannon B, Nedergaard J (2012). Recruited vs. nonrecruited molecular signatures of brown, “brite,” and white adipose tissues. *American Journal of Physiology*.

[50] Frontini A, Cinti S (2010). Distribution and development of brown adipocytes in the murine and human adipose organ. *Cell Metabolism*.

[51] Xue B, Rim JS, Hogan JC, Coulter AA, Koza RA, Kozak LP (2007). Genetic variability affects the development of brown adipocytes in white fat but not in interscapular brown fat. *Journal of Lipid Research*.

[52] Nedergaard J, Cannon B (2013). UCP1 mRNA does not produce heat. *Biochimica et Biophysica Acta*.

[53] Wu J, Cohen P, Spiegelman BM (2013). Adaptive thermogenesis in adipocytes: Is beige the new brown?. *Genes & Development*.

[54] Symonds ME, Bird JA, Clarke L, Gate JJ, Lomax MA (1995). Nutrition, temperature and homeostasis during perinatal development. *Experimental Physiology*.

[55] Aherne W, Hull D (1966). Brown adipose tissue and heat production in the newborn infant. *The Journal of Pathology and Bacteriology*.

[56] Alexander G, Bell AW (1975). Quantity and calculated oxygen consumption during summit metabolism of brown adipose tissue in newborn lambs. *Biology of the Neonate*.

[57] Hull D, Segall MM (1965). Heat production in the new-born rabbit and the fat content of the brown adipose tissue. *Journal of Physiology*.

[58] Clarke L, Heasman L, Firth K, Symonds ME (1997). Influence of route of delivery and ambient temperature on thermoregulation in newborn lambs. *American Journal of Physiology*.

[59] Widdowson EM (1950). Chemical composition of newly born mammals. *Nature*.

[60] Budge H, Symonds ME, Kurjak A, Chrervenak FA (2006). Fetal and neonatal nutrition—lipid and carbohydrate requirements and adaptations to altered supply at birth. *Textbook of Perinatal MEdicine*.

[61] Cannon B, Connoley E, Obregon M-J, Nedergaard J, Kunzel W, Jesen A (1988). Perinatal activation of brown adipose tissue. *The Endocrine Control of the Fetus*.

[62] Symonds ME, Stephenson T, Gardner DS, Budge H (2007). Long-term effects of nutritional programming of the embryo and fetus: mechanisms and critical windows. *Reproduction, Fertility and Development*.

[63] Symonds ME, Lomax MA (1992). Maternal and environmental influences on thermoregulation in the neonate. *Proceedings of the Nutrition Society*.

[64] Giralt M, Martin I, Iglesias R, Vinas O, Villarroya F, Mampel T (1990). Ontogeny and perinatal modulation of gene expression in rat brown adipose tissue. Unaltered iodothyronine 5′-deiodinase activity is necessary for the response to environmental temperature at birth. *European Journal of Biochemistry*.

[65] Blumberg MS, Sokoloff G (1998). Thermoregulatory competence and behavioral expression in the young of altricial species—revisited. *Developmental Psychobiology*.

[66] Symonds ME, Mostyn A, Stephenson T (2001). Cytokines and cytokine receptors in fetal growth and development. *Biochemical Society Transactions*.

[67] Clarke L, Bird JA, Lomax MA, Symonds ME (1996). Effect of *β*3-adrenergic agonist (Zeneca D7114) on thermoregulation in near-term lambs delivered by cesarean section. *Pediatric Research*.

[68] Trayhurn P, Temple NJ, Van Aerde J (1989). Evidence from immunoblotting studies on uncoupling protein that brown adipose tissue is not present in the domestic pig. *Canadian Journal of Physiology and Pharmacology*.

[69] Berg F, Gustafson U, Andersson L (2006). The uncoupling protein 1 gene (UCP1) is disrupted in the pig lineage: a genetic explanation for poor thermoregulation in piglets. *PLoS Genetics*.

[70] Pope M, Budge H, Symonds ME (2013). The developmental transition of ovine adipose tissue through early life. *Acta Physiologica Scandinavica*.

[71] Clarke L, Bryant MJ, Lomax MA, Symonds ME (1997). Maternal manipulation of brown adipose tissue and liver development in the ovine fetus during late gestation. *British Journal of Nutrition*.

[72] Gemmell RT, Bell AW, Alexander G (1972). Morphology of adipose cells in lambs at birth and during subsequent transition of brown to white adipose tissue in cold and in warm conditons. *American Journal of Anatomy*.

[73] Gemmell RT, Alexander G (1978). Ultrastructural development of adipose tissue in foetal sheep. *Australian Journal of Biological Sciences*.

[74] Scholzen T, Gerdes J (2000). The Ki-67 protein: from the known and the unknown. *Journal of Cellular Physiology*.

[75] Tseng YH, Kokkotou E, Schulz TJ (2008). New role of bone morphogenetic protein 7 in brown adipogenesis and energy expenditure. *Nature*.

[76] Kajimura S, Seale P, Kubota K (2009). Initiation of myoblast to brown fat switch by a PRDM16-C/EBP-*β* transcriptional complex. *Nature*.

[77] Bird JA, Spencer JAD, Mould T, Symonds ME (1996). Endocrine and metabolic adaptation following caesarean section or vaginal delivery. *Archives of Disease in Childhood*.

[78] Viengchareun S, Servel N, Fève B, Freemark M, Lombès M, Binart N (2008). Prolactin receptor signaling is essential for perinatal brown adipocyte function: a role for insulin-like growth factor-2. *PLoS ONE*.

[79] Pearce S, Budge H, Mostyn A (2005). Prolactin, the prolactin receptor and uncoupling protein abundance and function in adipose tissue during development in young sheep. *Journal of Endocrinology*.

[80] Ribeiro MO, Bianco SDC, Kaneshige M (2010). Expression of uncoupling protein 1 in mouse brown adipose tissue is thyroid hormone receptor-*β* isoform specific and required for adaptive thermogenesis. *Endocrinology*.

[81] Hall JA, Ribich S, Christoffolete MA (2010). Absence of thyroid hormone activation during development underlies a permanent defect in adaptive thermogenesis. *Endocrinology*.

[82] Uldry M, Yang W, St-Pierre J, Lin J, Seale P, Spiegelman BM (2006). Complementary action of the PGC-1 coactivators in mitochondrial biogenesis and brown fat differentiation. *Cell Metabolism*.

[83] Lomax MA, Sadiq F, Karamanlidis G, Karamitri A, Trayhurn P, Hazlerigg DG (2007). Ontogenic loss of brown adipose tissue sensitivity to *β*-adrenergic stimulation in the ovine. *Endocrinology*.

[84] Fernandez-Marcos PJ, Auwerx J (2011). Regulation of PGC-1*α*, a nodal regulator of mitochondrial biogenesis. *The American Journal of Clinical Nutrition*.

[85] Bassett JM, Symonds ME (1998). *β*
_2_-agonist ritodrine, unlike natural catecholamines, activates thermogenesis prematurely in fetal sheep. *American Journal of Physiology*.

[86] Seale P (2010). Transcriptional control of brown adipocyte development and thermogenesis. *International Journal of Obesity*.

[87] Gregoire FM, Smas CM, Sul HS (1998). Understanding adipocyte differentiation. *Physiological Reviews*.

[88] Li P (2004). Cidea, brown fat and obesity. *Mechanisms of Ageing and Development*.

[89] Forner F, Kumar C, Luber CA, Fromme T, Klingenspor M, Mann M (2009). Proteome differences between brown and white fat mitochondria reveal specialized metabolic functions. *Cell Metabolism*.

[90] Pilegaard H, Ordway GA, Saltin B, Neufer PD (2000). Transcriptional regulation of gene expression in human skeletal muscle during recovery from exercise. *American Journal of Physiology*.

[91] Clarke L, Buss DS, Juniper DT, Lomax MA, Symonds ME (1997). Adipose tissue development during early postnatal life in ewe-reared lambs. *Experimental Physiology*.

[92] Hallberg M, Morganstein DL, Kiskinis E (2008). A functional interaction between RIP140 and PGC-1*α* regulates the expression of the lipid droplet protein CIDEA. *Molecular and Cellular Biology*.

[93] Pan D, Fujimoto M, Lopes A, Wang YX (2009). Twist-1 is a PPARdelta-inducible, negative-feedback regulator of PGC-1alpha in brown fat metabolism. *Cell*.

[94] Schulz TJ, Tseng YH (2009). Emerging role of bone morphogenetic proteins in adipogenesis and energy metabolism. *Cytokine and Growth Factor Reviews*.

[95] Mostyn A, Pearce S, Budge H (2003). Influence of cortisol on adipose tissue development in the fetal sheep during late gestation. *Journal of Endocrinology*.

[96] Gnanalingham MG, Mostyn A, Symonds ME, Stephenson T (2005). Ontogeny and nutritional programming of adiposity in sheep: potential role of glucocorticoid action and uncoupling protein-2. *American Journal of Physiology*.

[97] Symonds ME, Mostyn A, Pearce S, Budge H, Stephenson T (2003). Endocrine and nutritional regulation of fetal adipose tissue development. *Journal of Endocrinology*.

[98] Symonds ME (1995). Pregnancy, parturition and neonatal development—interactions between nutrition and thyroid hormones. *Proceedings of the Nutrition Society*.

[99] Symonds ME, Bird JA, Sullivan C, Wilson V, Clarke L, Stephenson T (2000). Effect of delivery temperature on endocrine stimulation of thermoregulation in lambs born by cesarean section. *Journal of Applied Physiology*.

[100] Heasman L, Clarke L, Symonds ME (2000). Influence of thyrotropin-releasing hormone administration at birth on thermoregulation in lambs delivered by cesarean. *American Journal of Obstetrics and Gynecology*.

[101] Clarke L, Heasman L, Symonds ME (1998). Influence of maternal dexamethasone administration on thermoregulation in lambs delivered by caesarean section. *Journal of Endocrinology*.

[102] Mostyn A, Bispham J, Pearce S (2002). Differential effects of leptin on thermoregulation and uncoupling protein abundance in the neonatal lamb. *The FASEB Journal*.

[103] Bispham J, Budge H, Mostyn A (2002). Ambient temperature, maternal dexamethasone, and postnatal ontogeny of leptin in the neonatal lamb. *Pediatric Research*.

[104] Symonds ME, Andrews DC, Johnson P (1989). The control of thermoregulation in the developing lamb during slow wave sleep. *Journal of Developmental Physiology*.

[105] Clarke L, Darby CJ, Lomax MA, Symonds ME (1994). Effect of ambient temperature during 1st day of life on thermoregulation in lambs delivered by cesarean section. *Journal of Applied Physiology*.

[106] Kozak LP, Koza RA, Anunciado-Koza R (2010). Brown fat thermogenesis and body weight regulation in mice: relevance to humans. *International Journal of Obesity*.

[107] Gunn TR, Gluckman PD (1995). Perinatal thermogenesis. *Early Human Development*.

[108] Rudolph AM (1985). Distribution and regulation of blood flow in the fetal and neonatal lamb. *Circulation Research*.

[109] Lossec G, Lebreton Y, Hulin JC, Fillaut M, Herpin P (1998). Age-related changes in oxygen and nutrient uptake by hindquarters in newborn pigs during cold-induced shivering. *Experimental Physiology*.

[110] Alexander G, Williams D (1968). Shivering and non-shivering therogenesis during summit metabolism in young lambs. *Journal of Physiology*.

[111] Bal NC, Maurya SK, Sopariwala DH (2012). Sarcolipin is a newly identified regulator of muscle-based thermogenesis in mammals. *Nature Medicine*.

[112] Hondares E, Rosell M, Gonzalez FJ, Giralt M, Iglesias R, Villarroya F (2010). Hepatic FGF21 expression is induced at birth via PPARalpha in response to milk intake and contributes to thermogenic activation of neonatal brown fat. *Cell Metabolism*.

[113] Fisher FM, Kleiner S, Douris N (2012). FGF21 regulates PGC-1alpha and browning of white adipose tissues in adaptive thermogenesis. *Genes & Development*.

[114] Nedergaard J, Matthias A, Golozoubova V, Jacobsson A, Cannon B (1999). UCP1: the original uncoupling protein—and perhaps the only one?. *Journal of Bioenergetics and Biomembranes*.

[115] Symonds ME, Andrews DC, Johnson P (1989). The endocrine and metabolic response to feeding in the developing lamb. *Journal of Endocrinology*.

[116] Symonds ME, Bryant MJ, Clarke L, Darby CJ, Lomax MA (1992). Effect of maternal cold exposure on brown adipose tissue and thermogenesis in the neonatal lamb. *Journal of Physiology*.

[117] Schermer SJ, Bird JA, Lomax MA, Shepherd DAL, Symonds ME (1996). Effect of fetal thyroidectomy on brown adipose tissue and thermoregulation in newborn lambs. *Reproduction, Fertility and Development*.

[118] Symonds ME, Andrews DC, Buss DS, Clarke L, Darby CJ, Lomax MA (1996). Effect of rearing temperature on perirenal adipose tissue development and thermoregulation following methimazole treatment of postnatal lambs. *Experimental Physiology*.

[119] Darby CJ, Clarke L, Lomax MA, Symonds ME (1996). Brown adipose tissue and liver development during early postnatal life in hand-reared and ewe-reared lambs. *Reproduction, Fertility and Development*.

[120] Mostyn A, Symonds ME (2009). Early programming of adipose tissue function: a large-animal perspective. *Proceedings of the Nutrition Society*.

[121] Symonds ME, Andrews DC, Buss DS, Clarke L, Lomax MA (1996). Influence of rearing temperature on lung development following methimazole treatment of postnatal lambs. *Experimental Physiology*.

[122] Heaton JM (1972). The distribution of brown adipose tissue in the human. *Journal of Anatomy*.

[123] Gilsanz V, Chung SA, Jackson H, Dorey FJ, Hu HH (2011). Functional brown adipose tissue is related to muscle volume in children and adolescents. *Journal of Pediatrics*.

[124] Zhang C, McFarlane C, Lokireddy S (2012). Inhibition of myostatin protects against diet-induced obesity by enhancing fatty acid oxidation and promoting a brown adipose phenotype in mice. *Diabetologia*.

[125] Bostrom P, Wu J, Jedrychowski MP (2012). A PGC1-alpha-dependent myokine that drives brown-fat-like development of white fat and thermogenesis. *Nature*.

[126] Timmons JA, Baar K, Davidsen PK, Atherton PJ (2012). Is irisin a human exercise gene?. *Nature*.

[127] Ricquier D, Bouillaud F (2000). The uncoupling protein homologues: UCP1, UCP2, UCP3, StUCP and AtUCP. *Biochemical Journal*.

[128] Mostyn A, Litten JC, Perkins KS (2004). Influence of genotype on the differential ontogeny of uncoupling protein 2 and 3 in subcutaneous adipose tissue and muscle in neonatal pigs. *Journal of Endocrinology*.

[129] Clapham JC, Arch JRS, Chapman H (2000). Mice overexpressing human uncoupling protein-3 in skeletal muscle are hyperphagic and lean. *Nature*.

[130] Grueter CE, van Rooij E, Johnson BA (2012). A cardiac microRNA governs systemic energy homeostasis by regulation of MED13. *Cell*.

[131] Ojha S, Robinson L, Yazdani M, Symonds ME, Budge H Brown adipose tissue genes in pericardial adipose tissue of newborn sheep are downregulated by maternal nutrient restriction in late gestation.

[132] Budge H, Edwards LJ, McMillen IC (2004). Nutritional manipulation of fetal adipose tissue deposition and uncoupling protein 1 messenger RNA abundance in the sheep: differential effects of timing and duration. *Biology of Reproduction*.

[133] Budge H, Bispham J, Dandrea J (2000). Effect of maternal nutrition on brown adipose tissue and its prolactin receptor status in the fetal lamb. *Pediatric Research*.

[134] Mostyn A, Wilson V, Dandrea J (2003). Ontogeny and nutritional manipulation of mitochondrial protein abundance in adipose tissue and the lungs of postnatal sheep. *British Journal of Nutrition*.

[135] Svensson PA, Jernas M, Sjoholm K (2011). Gene expression in human brown adipose tissue. *International Journal of Molecular Medicine*.

[136] Sacks HS, Fain JN, Holman B (2009). Uncoupling protein-1 and related messenger ribonucleic acids in human epicardial and other adipose tissues: epicardial fat functioning as brown fat. *Journal of Clinical Endocrinology and Metabolism*.

[137] Ding J, Hsu FC, Harris TB (2009). The association of pericardial fat with incident coronary heart disease: The Multi-Ethnic Study of Atherosclerosis (MESA). *The American Journal of Clinical Nutrition*.

[138] Bartelt A, Bruns OT, Reimer R (2011). Brown adipose tissue activity controls triglyceride clearance. *Nature Medicine*.

[139] Chan LLY, Sébert SP, Hyatt MA (2009). Effect of maternal nutrient restriction from early to midgestation on cardiac function and metabolism after adolescent-onset obesity. *American Journal of Physiology*.

[140] Whittle AJ, Vidal-Puig A (2012). NPs—heart hormones that regulate brown fat?. *The Journal of Clinical Investigation*.

[141] Bordicchia M, Liu D, Amri EZ (2012). Cardiac natriuretic peptides act via p38 MAPK to induce the brown fat thermogenic program in mouse and human adipocytes. *The Journal of Clinical Investigation*.

[142] Symonds ME, Pope M, Sharkey D, Budge H (2012). Adipose tissue and fetal programming. *Diabetologia*.

[143] Rothwell NJ, Stock MJ (1983). Luxuskonsumption, diet-induced thermogenesis and brown fat: the case in favour. *Clinical Science*.

[144] Sacks H, Symonds ME Anatomical locations of human brown adipose tissue: functional relevance and implications in obesity and type 2 diabetes.

[145] Elqatni M, Ghafir D (2012). Images in clinical medicine. Hibernoma of the neck. *The New England Journal of Medicine*.

[146] Vijgen GH, Bouvy ND, Smidt M, Kooreman L, Schaart G, van Marken Lichtenbelt W (2012). Hibernoma with metabolic impact?. *BMJ Case Reports*.

[147] Bird JA, Mostyn A, Clarke L (2001). Effect of postnatal age and a *β*3-adrenergic agonist (Zeneca D7114) administration on uncoupling protein-1 abundance in the lamb. *Experimental Physiology*.

[148] Kozak LP (2010). Brown fat and the myth of diet-induced thermogenesis. *Cell Metabolism*.

[149] Fueger BJ, Czernin J, Hildebrandt I (2006). Impact of animal handling on the results of 18F-FDG PET studies in mice. *Journal of Nuclear Medicine*.

[150] Symonds ME, Pope M, Budge H (2012). Adipose tissue development during early life: novel insights into energy balance from small and large mammals. *Proceedings of the Nutrition Society*.

[151] Scazzina F, Del Rio D, Benini L (2010). The effect of breakfasts varying in glycemic index and glycemic load on dietary induced thermogenesis and respiratory quotient. *Nutrition, Metabolism & Cardiovascular Diseases*.

[152] Westerterp KR, Wilson SAJ, Rolland V (1999). Diet induced thermogenesis measured over 24 h in a respiration chamber: effect of diet composition. *International Journal of Obesity*.

[153] Yoneshiro T, Aita S, Kawai Y, Iwanaga T, Saito M (2012). Nonpungent capsaicin analogs (capsinoids) increase energy expenditure through the activation of brown adipose tissue in humans. *The American Journal of Clinical Nutrition*.

[154] Cypess AM, Chen YC, Sze C (2012). Cold but not sympathomimetics activates human brown adipose tissue in vivo. * Proceedings of the National Academy of Sciences of the United States of America*.

[155] Vosselman MJ, van der Lans AA, Brans B (2012). Systemic beta-adrenergic stimulation of thermogenesis is not accompanied by brown adipose tissue activity in humans. *Diabetes*.

[156] Saggerson ED, McAllister TWJ, Baht HS (1988). Lipogenesis in rat brown adipocytes. Effects of insulin and noradrenaline, contributions from glucose and lactate as precursors and comparisons with white adipocytes. *Biochemical Journal*.

[157] Carey AL, Formosa MF, Every B (2013). Ephedrine activates brown adipose tissue in lean but not obese humans. *Diabetologia*.

[158] Yoneshiro T, Ogawa T, Okamoto N (2012). Impact of UCP1 and beta3AR gene polymorphisms on age-related changes in brown adipose tissue and adiposity in humans.

[159] Forrest RH, Hickford JGH, Frampton CM (2007). Polymorphism at the ovine *β*-3-adrenergic receptor locus (ADRB3) and its association with lamb mortality. *Journal of Animal Science*.

